# Selenium Level in Patients with Vitiligo: A Meta-Analysis

**DOI:** 10.1155/2020/7580939

**Published:** 2020-06-11

**Authors:** Ting Dai, Sun Xiaoying, Xin Li, Li Hongjin, Zhou Yaqiong, Liang Bo

**Affiliations:** ^1^Department of Dermatology, Children's Hospital of Fudan University, National Children's Medical Center, Shanghai, China; ^2^Institution of Dermatology, Yueyang Hospital of Integrated Traditional Chinese and Western Medicine, Shanghai University of Traditional Chinese Medicine, Shanghai, China; ^3^Department of Dermatology and Venereology, The First Affiliated Hospital, Anhui Medical University, Hefei, Anhui, China

## Abstract

**Background:**

It has been reported that deficiency of selenium can cause autoimmune disease. This meta-analysis was aimed at evaluating whether there exits an association between selenium level and vitiligo.

**Methods:**

A comprehensive search was conducted on PubMed, Embase, China National Knowledge Infrastructure (CNKI), Wanfang Med Online, and China VIP databases from the inception to February 12, 2019. The main outcome was the standardized mean difference (SMD) with 95% confidence interval (CI) in serum selenium level between vitiligo patients and healthy controls.

**Results:**

A total of 8 studies with 305 vitiligo patients and 6156 healthy controls were included in this meta-analysis. The results showed that there was no significant difference in selenium level between vitiligo patients and healthy controls (SMD = 0.481, 95%CI = −0.642 to 1.604, *Z* = 0.840, *P* > 0.05). Further subgroup analysis stratified by area revealed that Asian vitiligo patients had decreased selenium level, while that finding was not observed in Caucasian patients (Asian: SMD = −0.303, 95%CI = −0.603 to −0.004, *P* < 0.05; Caucasian: SMD = 0.957, 95%CI = −0.752 to 2.665, *P* > 0.05).

**Conclusions:**

Although overall selenium level was similar between vitiligo patients and health controls, subgroup analysis showed decreased levels of selenium in Asian vitiligo patients. It may suggest a clinical tailored administration of selenium supplementation in Asian vitiligo patients.

## 1. Introduction

Vitiligo is an acquired disease characterized by progressive loss of melanocytes [1, 2]. This disease has been estimated to affect about 0.5-4% of the world population, making it the most common depigmentation disorder [3–5]. Vitiligo significantly affects the psyche of a patient and his/her interpersonal relationships. Multiple mechanisms have been implicated in the intrinsic and extrinsic defects of melanocytes, including genetics, oxidative stress, environment, metabolic abnormalities, innate immunity, and adaptive immunity [6, 7].

Several previous studies have suggested that oxidative stress contributes to the etiology of vitiligo [8–11]. These findings demonstrated that melanocyte was more susceptible to oxidative stress as the increased production of free toxic radicals can cause damage to cells. In addition, the increased H_2_O_2_ levels and declined catalase levels were found in vitiligo lesions [10, 11].

Selenium is a kind of important nutrient and trace mineral to human health, which appeared in more than 30 selenoproteins in humans [12]. It can influence immune cells or receptors, and the immune system is capable of mitigating the stressors [13, 14]. Selenium is vital to the function of glutathione peroxidase (GPx) isoenzyme, and through cascading enzyme systems, it can play a pivotal role in redox regulation. Deficiency of selenium may predispose to several skin diseases. There is evidence that selenium status is depressed and is related to the severity of long-lasting psoriasis [15]. Selenium nanoparticles exhibited significant anti-inflammatory activity in an arthritic rat model [16]. Picardo et al. reported that a therapeutic approach, containing different antioxidants, is beneficial for depigmentation in vitiligo patients. Therefore, selenium may play a protective role against vitiligo. Selenium supplementation is extensively used, although its excessive application may result in the increase of selenium toxicity.

Several reports have investigated the selenium level in vitiligo; however, their results were controversial; some showed an elevated level, whereas others reported no different or reduced level [17–19]. The difference could be related to variation in the duration or activity of vitiligo or various samples and populations. For instance, a number of scholars have previously reported the decrease of selenium level in Polish individuals, which may be caused by the low selenium content in dietary intake [20]. Thus, we applied a meta-analysis of previous reports concerning selenium level to indicate the existence of an association between selenium level and vitiligo.

## 2. Materials and Methods

### 2.1. Search Strategy

A comprehensive literature review was conducted in PubMed, Embase, China National Knowledge Infrastructure (CNKI), Wanfang Med Online, and China VIP databases from the inception to February 12, 2019. The following main search terms were used: “selenium concentration OR selenium content OR selenium level OR selenium” AND “vitiligo”. Additional potentially relevant studies were further manually searched from references listed in the original reports.

### 2.2. Study Selection

This meta-analysis was undertaken on the basis of analyzing all case-control studies that compared serum, plasma, or whole blood selenium levels. The inclusion criteria were as follows: (1) adult vitiligo patients, including localized, generalized, or universal vitiligo and stable or active vitiligo; (2) control group, involving healthy individuals; and (3) reported quantitative selenium level (mean ± standard deviation (SD)). Studies were excluded if (1) they included case report, comments, letters, or review; (2) they involved animal or in vitro experiments; and (3) it is duplicate publication.

### 2.3. Data Extraction

Two authors evaluated the titles and abstracts of the identified studies independently. Potentially related studies identified in the screening were further assessed. Data concerning the first author's name, year of publication, country, skin type, type and stage of vitiligo, sample size, age, type of sample, analysis method of selenium, and selenium level were extracted independently. If there were some discrepancies, they would reach a consensus through discussion.

### 2.4. Quality Assessment

We used the Newcastle-Ottawa Scale (NOS) system to estimate the quality of the included studies [21]. The NOS system was defined with three components: (1) selection of study groups, (2) comparability of groups, and (3) ascertainment of exposure and outcomes for case-control and cohort studies, respectively. Added scores ranged from 0 to 9 points (from the lowest to the highest). Any disagreements about the methodological quality of the results were resolved by discussion or by a third reviewer.

### 2.5. Statistical Analysis

Comprehensive meta-analysis 2.0 software (CMA; Biostat, Inc., Englewood, NJ, USA) was used to perform the statistical analysis. Standard error of the mean (SEM) was converted to SD. The standard mean difference (SMD) and corresponding 95% confidence interval (CI) were calculated using the original studies. The significance of pooled SMD was determined by the *Z*-test. The heterogeneity of size effect between the included publications was analyzed using the inconsistency index (*I*^2^) test and *Q*-statistic as described previously [22]. *I*^2^ values of 0.25, 0.50, and 0.75 showed small, moderate, and high levels of heterogeneity, respectively. If significant heterogeneity was observed, then a random-effects model would be used. Publication bias was assessed by Begg's test using the funnel plot [23].

Subgroup meta-analyses were conducted according to race (Caucasian and Asian) and the source of the sample (serum, plasma, and blood). Sensitivity analyses were carried out by sequentially removing one single article to examine the robustness of uncertainty in the meta-analysis.

## 3. Results

### 3.1. Eligible Studies

**Figure 1:**
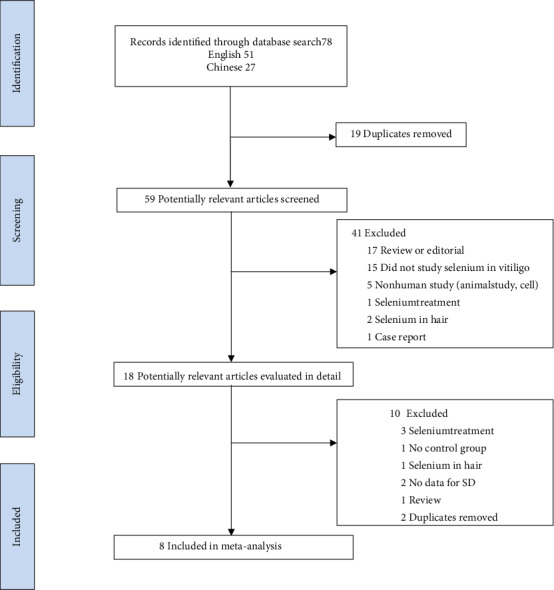
Flowchart of the screened and included studies.

Of the 78 relevant articles initially identified, 51 were in English and 27 in Chinese. Among them, 19 were excluded due to duplicate publication and 59 articles were included for further evaluation. In addition, 41 studies unrelated to selenium or vitiligo were excluded after evaluation of the titles and abstracts. After further analysis of the remaining 18 studies, 1 review, 2 duplicated studies, 1 study without a control group, 3 studies with inappropriate data, 2 studies with missing SD data, and 1 study only including selenium level were excluded. Eventually, 8 publications were included in this meta-analysis (see Figure 1) [17–19, 24–28].

### 3.2. Study Characteristics

All those publications were single-center studies that included vitiligo patients and healthy controls. The extracted data related to selenium level in healthy controls and vitiligo patients are shown in Table 1. According to the NOS system, the overall quality of the study ranged from 4 to 7 points. The 8 studies reported data belonging to 6361 subjects, including 305 patients with vitiligo and 6156 health controls. Of the 8 studies, the sample resource was either serum, plasma, or blood. The race of the subjects in the included studies was Caucasian or Asian. Table 1 shows the characteristics of the included studies.

### 3.3. Overall Meta-Analysis

Among those 8 studies that measured the selenium level, 4 studies [17, 26–28] showed a similar selenium level between the vitiligo patients and healthy controls; 2 studies reported significantly elevated level while two studies expressed decreased selenium level in the vitiligo patients [18, 19, 24, 25]. Since the heterogeneity test indicated high statistical heterogeneity (*Q* = 303.1, *P* < 0.01; *I*^2^ = 97.7%), a random-effects model was used for the meta-analysis.

**Figure 2:**
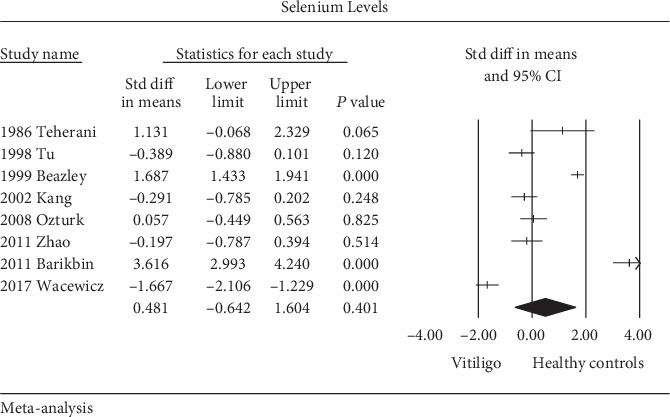
Forest plot for the random-effects model in meta-analysis. Selenium level in patients with vitiligo and healthy controls.

The results indicated that vitiligo patients had similar selenium levels compared with healthy controls (SMD = 0.481, 95%CI = −0.642 to 1.604, *Z* = 0.840, *P* > 0.05) (Figure 2).

### 3.4. Subgroup Analysis

When subgroup analysis was stratified by sample sources, there was no significant difference in selenium level in the vitiligo group in plasma (SMD = 0.057, 95%CI = −0.449 to 0.563, *P* > 0.05), blood (SMD = 1.131, 95%CI = −0.086 to 2.329, *P* > 0.05), and serum (SMD = 0.456, 95%CI = −0.949 to 1.860, *P* > 0.05) (Table 2). Further subgroup analysis stratified by area found that Asian vitiligo patients had decreased selenium levels compared with healthy controls (Asian: SMD = −0.303, 95%CI = −0.603 to −0.004, *P* < 0.05); however, Caucasian individuals did not exhibit any significant difference in selenium level (Caucasian: SMD = 0.957, 95%CI = −0.752 to 2.665, *P* > 0.05).

### 3.5. Sensitivity Analysis and Publication Bias

**Figure 3:**
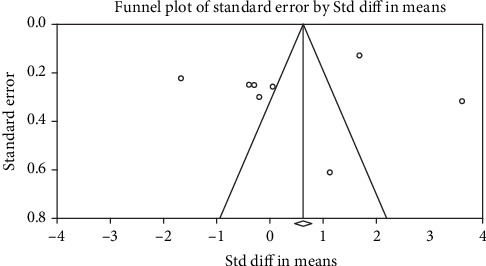
Funnel plots in the studies reporting selenium level in vitiligo patients and healthy controls.

We conducted the funnel plot to assess potential publication bias. Begg's test was used to investigate the possibility of publication bias by using CMA 2.0 software. Begg's test did not indicate any significant publication bias for selenium level (Kendall′s tau = 0.357, two-tailed *P* = 0.27) (Figure 3).

The results of the sensitivity analysis showed minor changes in SMD and 95% CIs when one study was removed from the meta-analysis, demonstrating that no individual study had a remarkable influence on the overall outcomes.

## 4. Discussion

Several different factors may contribute to the destruction of melanocytes in vitiligo, and the most important hypothesis includes genetics, altered redox status, autoimmunity, and self-destruction.

Selenium is deemed to be a vital trace element, and it is an essential component for numerous enzymes, such as selenoprotein P, GPx, and formate dehydrogenase. In addition, it acts as an antioxidant and immunomodulatory agent, influencing specific immune pathways in both humans and animals. Huang et al. reported that selenium level could affect T cell differentiation, and deficiency of selenium is associated with Th2 cells/markers [29]. Inorganic selenium-based salts can inhibit the production of some inflammatory cytokines in vitro, including tumor necrosis factor-*α* (TNF-*α*), interleukin-1*α* (IL-1*α*), and interleukin-6 (IL-6) [30]. As selenocysteine, it can reduce the possibility of oxidative damage to lipoproteins, lipids, and DNA. Sufficient selenium intake and its usage are important for normal immunity [31]. In some immune or inflammatory skin patients, skin cancer and malignant melanoma, low concentration of selenium, and depressed selenium-dependent enzyme activity were observed [13]. Selenium supplementation may contribute to the prevention of immune-mediated thyroid disorders [32]. Based on a previous study, selenium supplementation contributed to superior clinical efficacy to treat vitiligo [33]. In another study, decreased selenium level in vitiligo was observed after phototherapy. Selenium consumption in vitiligo might contribute to the decrease of selenium level.

Reported levels of selenium in patients with vitiligo were controversial. To date, no meta-analysis has analyzed the selenium level among vitiligo patients. The current meta-analysis investigated whether selenium level is of great importance in the development of vitiligo. Herein, the overall pooled selenium level was similar between vitiligo patients and healthy controls. As there was statistical heterogeneity among studies, we further conducted a subgroup analysis. The subgroup analysis unveiled that selenium level was lower in vitiligo patients than in health controls in the Asian population, while no significant difference was found between Caucasian individuals and healthy controls. These results suggested that low selenium level may contribute to the pathogenesis of vitiligo in the Asian population, unlike the Caucasian people. This is similar to the previous pooled GPx level in vitiligo patients, although the overall GPx level was the same between vitiligo patients and healthy controls; however, subgroup analysis uncovered that the GPx level was lower than that in healthy controls in the Asian population and segmental vitiligo patients [34]. Previous metaregression about GPx showed that race could be the major source of the heterogeneity. Selenium is a structural component of the GPx enzyme, and race could also be the reason for low selenium in the Asian population. On the other hand, selenium intake differs significantly among different areas. Correlation between selenium concentration and GPx activity was found only if the selenium intake was below the recommended amount. Therefore, we speculated that selenium supplementation might be helpful to treat vitiligo in the Asian population.

When subgroup analysis was stratified by sample, vitiligo patients showed similar selenium levels compared with healthy controls in blood, plasma, and serum. Several biological samples such as hair, toenails, whole blood, serum, plasma, CSF, and urine were used to assess the selenium status. Serum/plasma was the most widely used circulating markers. A previous small study showed no significant differences in the selenium concentrations between serum and plasma either.

The current meta-analysis contains several limitations. Firstly, there was a significant inter study heterogeneity across the included studies, which may be related to vitiligo stage and type, racial, and gender factors or selenium analysis method. Secondly, the number of participants in this meta-analysis was relatively limited, in that only 6361 participants from 8 studies were included. The number of participants included in the subgroup analysis was even smaller. Therefore, further studies with larger sample size should be conducted to provide more promising results. Finally, for observational studies, we were unable to adjust for potential confounding factors in patients' baseline characteristics that were related to selenium levels.

## 5. Conclusion

Although this meta-analysis demonstrated that the overall selenium level was similar between vitiligo patients and health controls, subgroup analysis showed decreased levels of selenium in Asian vitiligo patients. The results may suggest a clinical tailored use of selenium supplementation in Asian vitiligo patients. Further studies with larger sample size should be conducted to provide more promising data.

## Figures and Tables

**Table 1:** Characteristics of the 8 studies included for meta-analysis.

Study/year	Country	Skin type	Sample	Age, mean ± SD (range)	Vitiligo/control	Type/stage	Case	Control	Uni/methods	NOS score
Teherani and Nagy-Vezekenyi [24]	Austria	NA	Blood	(9-16)	5/8	10-25% body surface	0.388 ± 0.044	0.304 ± 0.087	*μ*g/g dry weight neutron activation analysis	4
Tu et al. [26]	China	NA	Serum	29.1 (18-51)	29/37	Localized 13 Segmental 5 Generalized 11	99.41 ± 14.93	105.24 ± 14.92	*μ*g/L fluorescence spectrophotometry	7
Beazley et al. [19]	Germany	II (n.5) III (n.53) IV (n.3)	Serum	38.9 (12 ± 63)	61/5932	Vulgaris 44 Acrofacial 13 Segmental 2 Focal form 1 Totalis 1 (2 active stages)	1.27 ± 0.32	0.93 ± 0.20	*μ*mol/L atomic absorption spectrophotometry	6
Kang et al. [27]	China	Dark-skinned patients	Serum	(3-64)	30/34	3 m-30 y	0.10 ± 0.02	0.13 ± 0.04	mg/L	5
Ozturk et al. [25]	Turkey	NA	Plasma	23.6 ± 7.4 (16-43)	30/30	Generalized Stable	122.33 ± 30.17	120.77 ± 21.80	Ppb fluorescence spectrophotometer	7
Barikbin et al. [17]	Iran	III or IV	Serum	31.8 (9-82)	60/45	Vulgaris	1.021 ± 0.31	0.909 ± 0.07	*μ*mol/L flameless atomic absorption method	6
Zhao et al. [28]	China	II	Serum	(3-65)	36/16	Active 18/stable 18 Localized 16 Generalized 17 Scattered 2 Segmental 1	121.9 ± 46.16	129.27 ± 23.67	ng/L	6
Wacewicz et al. [18]	Poland	NA	Serum	44.8 ± 15.6 (21-73)	50 + 58	Generalized	51.30 ± 13.99	79.42 ± 18.97	*μ*g/L electrothermal atomic absorption spectrometry method	7

**Table 2:** Subgroup analysis of selenium level.

	SMD	Lower limit	Upper limit	*P* value
Area				
Asia	-0.303	-0.603	−0.004	0.047
Caucasian	0.957	-0.752	2.665	0.272
Sample				
Blood	1.131	-0.086	2.329	0.065
Plasma	0.057	0.449	0.563	0.825
Serum	0.456	-0.949	1.860	0.525
